# Gut Microbial Metabolome and Dysbiosis in Neurodegenerative Diseases: Psychobiotics and Fecal Microbiota Transplantation as a Therapeutic Approach—A Comprehensive Narrative Review

**DOI:** 10.3390/ijms241713294

**Published:** 2023-08-27

**Authors:** Sara Uceda, Víctor Echeverry-Alzate, Manuel Reiriz-Rojas, Esther Martínez-Miguel, Ana Pérez-Curiel, Silvia Gómez-Senent, Ana Isabel Beltrán-Velasco

**Affiliations:** 1 BRABE Group, Psychology Department, School of Life and Nature Sciences, Nebrija University, 28240 Madrid, Spain; 2 Health Department, School of Life and Nature Sciences, Nebrija University, 28240 Madrid, Spain; 3 Psychology Department, School of Life and Nature Sciences, Nebrija University, 28240 Madrid, Spain

**Keywords:** neurodegenerative diseases, microbiota, dysbiosis, metabolites, interventions, prebiotics, probiotics, psychobiotics, fecal microbiota transplantation

## Abstract

The comprehensive narrative review conducted in this study delves into the mechanisms of communication and action at the molecular level in the human organism. The review addresses the complex mechanism involved in the microbiota–gut–brain axis as well as the implications of alterations in the microbial composition of patients with neurodegenerative diseases. The pathophysiology of neurodegenerative diseases with neuronal loss or death is analyzed, as well as the mechanisms of action of the main metabolites involved in the bidirectional communication through the microbiota–gut–brain axis. In addition, interventions targeting gut microbiota restructuring through fecal microbiota transplantation and the use of psychobiotics—pre- and pro-biotics—are evaluated as an opportunity to reduce the symptomatology associated with neurodegeneration in these pathologies. This review provides valuable information and facilitates a better understanding of the neurobiological mechanisms to be addressed in the treatment of neurodegenerative diseases.

## 1. Introduction

### 1.1. Microbiota and Neurodegeneration

Within the classification of neurodegenerative diseases, we find at least 100 different pathologies, which present specific symptomatology, with the most prevalent being Alzheimer’s disease (AD), Parkinson’s disease (PD), amyotrophic lateral sclerosis (ALS), and multiple sclerosis (MS). It is estimated that more than 55 million people are living with Alzheimer’s disease, and the prevalence of PD has significantly increased in recent decades, reaching nearly 9 million people affected worldwide. MS affects almost 3 million people worldwide. As for ALS, the incidence rate in the general population is 5/100,000 people worldwide. All these data indicate that the impact of neurodegenerative diseases has considerably increased, while the number of years with a reduced quality of life has also increased [1,2,3].

On the other hand, the understanding of the microbiota and its implication in different metabolic processes has received growing interest from the scientific community over the years, and specifically the study of these processes and its implication in neurodegenerative diseases. It is now known that the brain regulates intestinal functions in communication with the hypothalamic–pituitary–adrenal axis, as well as the autonomic nervous system (ANS) [4,5]. In addition, the intestine modulates certain functions in the central nervous system (CNS) involving various metabolites produced by the microbiota, as certain gut hormones and neuroactive compounds that propagate through the enteric nervous system (ENS), the circulatory system, the vagus nerve, or the immune system, until reaching the brain [6,7]. This chain of communication forms the so-called microbiota–gut–brain axis, where it is estimated that 10^13^–10^14^ live microorganisms exist. Thus, this microbiota–gut–brain axis is a bidirectional communication pathway consisting of neuronal, neuroimmune, endocrine, and metabolic signals [8,9,10].

In this regard, it is known that alterations in the composition of the microbiota are associated with multiple pathologies, including obesity, certain mental disorders, CNS disorders, gastric disorders, as well as neurodegenerative diseases, with very specific microbial profiles being found in these patients [11]. Microbial-derived substances circulate freely along the microbiota–gut–brain axis, allowing the passage of harmful substances and their propagation in different pathways and systems of the organism. Precisely this constant communication through this axis makes it possible to consider the microbiota as a treatment opportunity, a therapeutic target for intervention in neurodegenerative diseases, with the aim of protecting the gut and hindering the entry of pathogenic substances into the modulating axis [12].

Therefore, the main objectives of this comprehensive narrative review are: (i) to analyze the role of the gut microbiota (GM), its dysbiosis, and the microbial metabolome in the pathogenesis of the neurodegenerative diseases, and (ii) to explore the use of psychobiotics and fecal microbiota transplantation as a therapeutic approach.

### 1.2. Methodology of Search

In order to perform this literature review, different databases containing the most current information in this field of study were consulted, i.e., WoS (Web of Science), PubMed, ScienceDirect, Cochrane (Wiley), and Scopus. The search was performed using this article’s keywords: neurodegenerative diseases, microbiota, dysbiosis, metabolites, interventions, prebiotics, probiotics, psychobiotics, and fecal microbiota transplantation, in addition to the names of each pathology (Alzheimer’s disease, Parkinson’s disease, multiple sclerosis, or amyotrophic lateral sclerosis). The MeSH guidelines were followed, thus ensuring the adequacy of the literature consulted. The search period was limited to articles published between 2000 and 2023, which guarantees the timeliness and relevance of the information included in this review. Nevertheless, a very small number of papers prior to these dates were included, with the aim of providing a specific background and initial data on studies in this line of research.

The authors meticulously revised the titles and abstracts of all the articles consulted. Exclusion criteria were applied according to procedures of previous reviews [13,14,15]: (i) studies outside the period analyzed, (ii) topics presented outside the scope of the review, and (iii) books, conference proceedings, doctoral theses, and abstracts. All studies that complied with scientific methodological standards and relevant information from the subsections of the present review were used. Once the most appropriate articles were identified, the authors began to work individually to extract the most significant results. The treatment of the information was carried out by all the authors of the review and, finally, the selected articles were discussed in order to write the present narrative review, which allowed maintaining the rigor of the information, with a shared perspective. Precisely for this purpose, meetings were held in which the obtained findings were pooled and the information was discussed and synthesized. This teamwork process facilitated the integration of the information and guaranteed the coherence of the data in this study.

## 2. Microbiota–Gut–Brain Axis in Neurodegenerative Diseases: Role of Microbial Metabolome

### 2.1. Eubiosis and Dysbiosis

It is known that people have a very high number of microorganisms that form an essential ecosystem in the human organism [16]. It is estimated that there are around 100 million bacteria and more than 300 different species, with different functions that allow the organism to function properly [8,17]. Although there are several tissues that are ideal for microorganisms to develop, such as the skin, the oral cavity, or the vaginal tract, it is in the digestive tract where 95% of these bacteria are found [18]. They are essential for the correct absorption of nutrients and function as a defensive barrier of intestinal mucus. This facilitates the non-proliferation of pathogenic bacteria and/or viruses [19,20].

When there is an alteration of the intestinal flora, or even an elimination of it, we speak of dysbiosis [21]. This imbalance can be determined by inflammatory processes, autoimmune diseases, unhealthy diets that include high amounts of protein and low amounts of fiber, chronic stress situations, drug abuse, and pharmacological treatments, such as metformin, nonsteroidal anti-inflammatory drugs, opioids, and statins, among others [22,23,24]. It is important to highlight that the use of antibiotics modifies the microbiota of the organism, affecting not only gene expression and protein activity, but also altering the functioning of the endocrine, metabolic, and immune systems [25,26].

When dysbiosis appears, very heterogeneous symptoms can be observed, not always of a gastrointestinal nature [27]. Among the most frequent are muscular disorders, joint disorders, skin disorders, headaches and migraines, irritability, sleep disturbances, chronic fatigue, and digestive discomfort, such as acute episodes of diarrhea, stomach gas, abdominal bloating, steatorrhea, diverticulitis, vitamin B12 deficiency, and even irritable bowel syndrome [27,28].

If dysbiosis is suspected, it is necessary to collect fecal samples for analysis by stool culture to quantify the intestinal microbiota or flora [29]. If the patient has a healthy or preserved microbiota (eubiosis), adequate concentrations of the microorganisms *E. coli*, *Enterococcus* sp., *Lactobacillus* sp., *Bifidubacterium*, *Bacteroides* sp., and *Prevotella* sp. will be observed [30,31,32]. However, there are different types of dysbiosis, depending on the intestinal region affected, such as IMO (intestinal methanogen overgrowth), LIBO (large intestine bacterial overgrowth), SIFO (small intestine fungal overgrowth), and LIFO (large intestine fungal overgrowth), but all of them are known as SIBO (small intestinal bacterial overgrowth) [33]. In all the above, there is a common denominator: the migration of bacteria, for example, from the colon to the small intestine, which will facilitate the appearance of toxins and intestinal gas [33,34,35].

**Figure 1:**
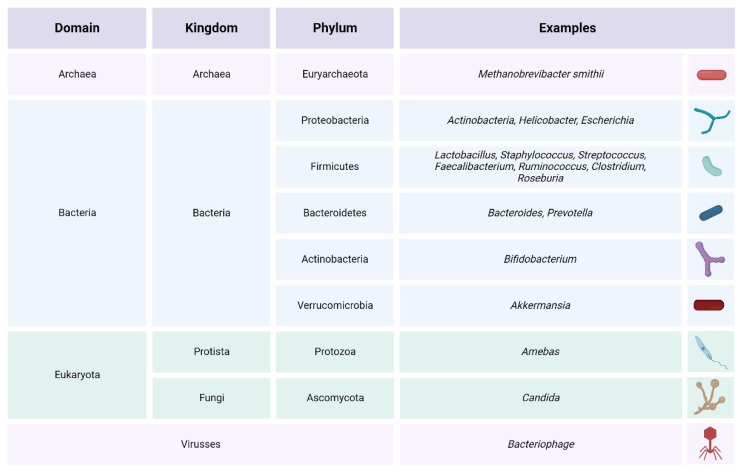
Gut microbiota composition. Principal gut microbial composition, including bacteria, archaea, eukaryota, and viruses.

In recent years, the study of the human microbiome has greatly advanced. This term refers to the set of microorganisms, as well as their genes and metabolites [36]. The same is true of metagenomics, which is the analysis of the genetic material of bacteria, making it possible to identify them through a biological sample [37]. These advances have allowed to identify 30% of the intestinal microbiota and its genes, although not all of them are culturable [38]. Thus, it is now known that the human gut microbiota community is formed by more than 1000 different species, which have a total of 150 times more genes than the human genome [39]. Within this great number of microorganisms that live in the human gut, bacteria from the *Firmicutes* and *Bacteroidetes* phyla, which together make up approximately 90% of the total, are the most notable. Other examples of bacteria present in the GM are the *Actinobacteria*, *Proteobacteria,* and *Verrucomicrobia* phyla. Besides the bacteria, the gut microbiota is comprised of other microorganisms such as archaea, viruses, fungi, and protozoa [40,41,42] (see Figure 1).

There are several elements that have an impact on the microbiota, with genetics and each person’s diet being determining factors [43]. Great advances have been made in recent decades in relation to the pathologies associated with alterations in the microbiota that allow determining the negative impact of this dysbiosis in different pathologies [44]. The microbiota is closely related to the response of the immune system, and it is also known that the decrease in the microbial load may be influenced by the hygienic habits of developed societies [45,46]. Similarly, diet is a key element in explaining this response when studying the incidence and prevalence of inflammatory diseases, such as type 1 diabetes, obesity, allergies, and asthma [47]. The so-called Western diet, rich in fat and sugars and low in fiber, produces changes in the microbiota [48], and recent studies have shown how this diet produces alterations in the microbial composition in mice, in a single day. These mice showed adiposity in two weeks, and alterations in metabolic pathways. Specifically, they showed an increase in *Firmicutes* and a decrease in *Bacteroidetes* [49,50]. On the other hand, the incorporation of fiber in the diet may be associated with the speed of intestinal transit since the same results were obtained by including osmotic laxatives in the diet [51].

Regarding immune and inflammatory responses, the microbiota performs adaptive functions through antigens called pathogen-associated molecular patterns (PAMPs), such as lipopolysaccharides (LPS), lipids, and lipoproteins [52]. The interaction between PAMPs and pattern recognition receptors favors the production of interferons and cytokines, which activate an immune response [53]. In this sense, it seems that the microbiota of children in developing countries and children in developed societies are different, justifying an increase in the diagnosis of pathologies associated with allergies and asthma in the latter, while there is no accumulation of PAMPs in the microbiota of children in developing countries [54].

### 2.2. Dysbiosis in Neurodegenerative Pathologies

The GM not only affects different diseases directly related with the digestive tract, as described above, but there is scientific evidence that associates the human microbiome with psychopathologies. For example, recent studies have shown that the gut microbiota is essential in the onset and maintenance of diseases that are related to the CNS, such as mood disorders, schizophrenia, or autism, and even with neurodegenerative disorders such as Alzheimer’s disease (AD), Parkinson’s disease (PD), multiple sclerosis (MS), amyotrophic lateral sclerosis (ALS), or Huntington´s disease, among others [55,56,57]. In addition, we now know that the certain markers of the pathology are related to an increase in intestinal permeability, called leaky gut syndrome [58].

#### 2.2.1. Alzheimer’s Disease

Alzheimer’s disease (AD) is the most prevalent form of dementia, and its onset is associated with an accumulation of β-amyloid and neurofibrillary tangles formed by phosphorylated Tau protein [59,60]. The relationship between mild behavioral impairment (MBI) and AD seems increasingly clear. Thus, MBI could be implicated at the onset of AD by different metabolic pathways and processes, such as low bacterial diversity, production of different harmful metabolites, direct activation of pro-inflammatory cytokines, or altered intestinal permeability. This allows different metabolites to cross the intestinal barrier and reach the brain, inducing neuroinflammation and brain and hippocampal dysfunction [61,62,63].

**Figure 2:**
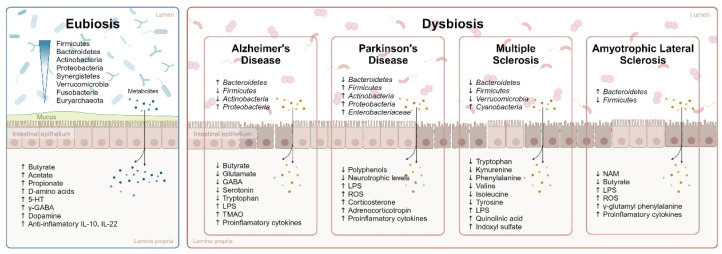
Dysbiosis in neurodegenerative pathologies. Main intestinal microbiota composition in eubiosis (from higher to lower abundance) [147] and representation of the main changes observed in the dysbiosis of neurodegenerative pathologies. Arrows represent increase or decrease compared to healthy controls, according to the literature. Abbreviations: 5-HT: 5-hydroxytryptamine (serotonin); GABA: gamma-aminobutyric acid; IL: interleukin; LPS: lipopolysaccharides; NAM: nicotinamide; ROS: reactive oxygen species; TMAO: trimethylamine-N-oxide.

Currently, there are many studies that highlight the relationship between different bacteria and AD. In this sense, patients with AD present an increase in *Proteobacteria* and *Bacteroidetes* phyla, together with a decrease in *Firmicutes* and *Actinobacteria* phyla [64,65,66]. For example, microorganisms such as *Chlamydia pneumoniae*, *Borrelia burgdorferi*, *Treponema pallidum*, *Helicobacter pylori*, *Escherichia coli*, *Escherichia-Shigella*, *spirochetes*, or *P. gingivalis* have been found in a higher proportion in the brain or cerebrospinal fluid (CSF) in patients with AD than in the general population, while the reduction of other species has been found in patients with AD, such as *Butyrivibrio*, *Eubacterium Clostridium* sp., *Roseburia hominis*, and *Faecalibacterium prausnitzii* [67]. Thus, a dysbiosis in the GM due to an increase in the pro-inflammatory bacteria and a decrease in the anti-inflammatory bacteria can be seen in AD, all of which produces an increase of different metabolites (e.g., LPS, trimethylamine-N-oxide, or pro-inflammatory cytokines) and a decrease of other metabolites, such as butyrate [68]. In this section, the main microbial metabolites implicated in the apparition of AD will be summarized, focusing on their action in the functioning of the central nervous system (see Figure 2).

##### Metabolites Altered in AD

- AD and Trimethylamine-N-oxide

Trimethylamine-N-oxide (TMAO) is a bacterial derivate metabolite of choline, l-carnitine, and betaine, among others, which is related to the AD apparition [69,70]. Thus, for example, patients with AD presented high levels of TMAO in their cerebrospinal fluid. Moreover, the presence of TMAO in this fluid demonstrates that this metabolite has the capacity to reach the CNS and, therefore, could affect its function [71].

The synthesis of TMAO requires the GM and the liver to intervene. Therefore, the first step is for the microbiota to synthesize trimethylamine (TMA) from choline, l-carnitine, or betaine. Lastly, the TMAO will be synthesized in the liver by the oxidation of TMA [72,73]. Some of the phyla with a high production of TMA could be *Clostridium*, *Escherichia*, and *Proteus* (*Firmicutes* and *Proteobacteria*), and a high presence of these bacteria could result in a high level of TMAO that could be related to dementia and AD [74,75].

A recent study by Vogt et al. (2018) analyzed this relation between TMAO and AD and found that the level of TMAO was elevated in patients with AD and associated with neuron death [69]. In this study, the authors analyzed TMAO levels in the CSF in 424 patients with AD and, using a comprehensive neuropsychological battery, they analyzed the patients’ cognitive status. They found that the level of TMAO was associated with higher AD dementia and cognitive impairment. Moreover, positive relations were found between TMAO levels and p-tau and p-tau/Aβ42, but not with amyloid biomarkers. In addition, they found that the TMAO seems to be related to the axon but not the dendritic injury due to the relation between TMAO and t-tau (axon injury), but not with neurofilament light chain (NFL, dendritic injury) [69,76].

The observed negative effect of TMAO could be explained by different mechanisms. In this sense, TMAO seems to alter hormonal and lipid homeostasis, cause platelet hyperreactivity, decrease cholesterol transport by changing sterol and cholesterol metabolisms, or activate the NLRP3 inflammasome, producing endothelial dysfunction. Additionally, it induces the release of inflammatory cytokines such as IL-1β and IL-18, produces neural senescence, causes oxidative stress, impairs mitochondrial function, inhibits mTOR signaling, induces CD68 expression (cellular marker associated with dementia), and upregulates macrophage scavenger receptors. Furthermore, in mice, TMAO is associated with atherosclerosis, which in turn is associated with dementia [69,77,78,79,80].

Finally, it should be noted that TMAO precursors, such as choline, have been linked to proper brain function, for example, through the reduction of homocysteine levels, which have neurotoxic properties [81]. In fact, an increase in choline levels (e.g., through diet) could present both benefits, due to choline function in the CNS, and disadvantages, due to TMAO synthesis. However, at least in young, healthy individuals, an increase in dietary TMAO precursors was not associated with an increase in plasma TMAO levels [81,82]. Therefore, although the relation between TMAO and cognitive impairment seems to be complex, the GM may play a key role [83].

- AD and Lipopolysaccharide

Another microbiota product that could be involved in AD is the Gram-negative membrane protein lipopolysaccharide (LPS), an endotoxin that produced inflammation in experimental animals [84]. LPS has been attributed pro-inflammatory properties and was found in patients with AD in higher levels compared to controls. Moreover, it has been found in the amyloid plaques and in vessels in patients with AD [85,86].

A related study carried out by Marizzoni et al. (2020) analyzed the possible relation between LPS with the amyloid pathology [87]. In this study, the LPS could be a risk factor in AD. The authors found that there was an association between elevated LPS levels in plasma and greater amyloid pathology in all cerebral regions studied. Other authors found how *P. gingivalis,* which secretes LPS (and causes periodontal disease in human), is associated not only with AD but also with a higher cerebral Aβ load [88,89]. Moreover, high levels of LPS were associated with higher blood levels of pro-inflammatory cytokines, such as IL-1β, NLRP3, CXCL2, and IL-18.

There are different mechanisms that could explain this effect of LPS in AD. For example, in in vitro experiments, LPS increased the Aβ fibrils’ formation, and when administered to rats (injected into the ventricles), it caused inflammation and other pathologies associated with AD [64]. In addition, LPS and Aβ increase the expression of ICAM-1 and PECAM-1, which modulate the trans-endothelial migration of leukocytes across the blood–brain barrier (BBB) and, therefore, could be the first step in the endothelial signaling cascade and accelerate neuroinflammation [90]. Thus, LPS provoked an inflammatory response. For example, the exposition of the human primary brain cells to LPS activates the transcription of the pro-inflammatory factor NF-κB (p50/p65) complex, triggering inflammatory neurodegeneration in the AD brain [91]. Finally, LPS joined with TLR4 receptors in the microglia produces an immune response similar to that observed in the microglia of AD patients [92].

It is important to highlight that some authors have analyzed the possible impact of SCFAs in AD, with controversial results. Traditionally, SCFAs have been proposed as metabolites with neuroprotective properties [93]. SCFAs could attenuate AD since these products could serve as a substrate for metabolic energy, inhibit histone deacetylases, induce the enteroendocrine signaling, activate the vagus nerve, have anti-inflammatory properties, and are involved in the correct function of the microglia in the brain, among other functions [94,95,96]. For example, propionate and butyrate inhibit histone deacetylase and, as a result, change the gene expression, which in the end reduced tumor formation and inflammation signaling. However, Marizzoni et al. (2020) found a positive relation between butyrate with the endothelial integrity; in fact, species which produced butyrate, such as *Clostridium butyricum,* improved AD symptoms [87,97]. In addition, the study showed a positive relation between acetate and valerate with the amyloid deposition. The authors hypothesized that acetate elevates the production of cytokines (IL-6, CXCL1, and CXCL2) in intestinal cells [98] and could have an important role in the amyloid aggregation and endothelial dysfunction. Therefore, considering these contradictory effects observed in different studies, the influence of each specific SCFA in AD should be further studied.

- AD and Amyloid

Some bacteria in the GM have the capacity to synthesize amyloid. These proteins have self-aggregation properties and cause cell deterioration when they accumulate [68,99]. Examples include *Escherichia coli* (curli), *Bacillus subtilis* (TasA), *Salmonella Typhimurium* (CsgA), *Pseudomonas fluorescens* (FapC), and *Staphylococcus aureus* (phenol-soluble modulins), among others. The amyloid allows bacteria to bind together and generate a biofilm that makes them more resistant to physical damage or immune attack [100]. These proteins have been associated with the misfolding of Aβ fibrils and other oligomers [68]. Thus, it seems that the amyloid produced by the GM is very similar to others present in the brain, at least in their tertiary form [101]. Therefore, bacterial amyloid could act as prion proteins and cause, through molecular mimicry, the proteins to adopt a pathogenic configuration (such as a β-sheet structure) [100]. In this sense, phenol-soluble modulins have cross-α structures and form cross-β fibrils that are associated with AD [102]. CsgA, which is a subunit of curli, is similar in shape to Aβ42 and induces plaque deposition in the brain [103]. Moreover, the authors found that the presence of some bacterial amyloid was associated with an increase in the immune system activation and in the pro-inflammatory cytokines’ (IL-17A, IL-22, IL-6, or TNF) production, and with a reduction of anti-inflammatory cytokines (IL-10) [104]. This effect could be provoked by the production of amyloid in the brain [104].

- AD and Neurotransmitters

Some GM bacteria have the ability to synthesize neurotransmitters associated with human physiology [105]. For example, *Escherichia* sp. synthesizes catecholamines such as norepinephrine [106], *Bacillus* sp. synthesizes dopamine and norepinephrine [107], and *Escherichia* spp. and *Lactobacillus* spp. produce GABA [108].

Glutamate is a neurotransmitter with a crucial role in cognition and CNS functioning. In fact, glutamate is the major excitatory neurotransmitter in mammalian CNS [109], and the disruption of normal glutamate signaling is associated with AD and many other neurodegenerative disorders (such as Parkinson’s disease, Huntington’s disease, multiple sclerosis, and schizophrenia, among others) [110,111]. Indeed, in patients with AD, the level of glutamate in cerebrospinal fluid is diminished [112], although the neurodegeneration observed in AD is associated with an increase in the glutamate level in the synapsis due to exocytosis. This glutamate effect seems to be mediated by the excessive Ca^2+^ entry in the cell [113,114].

Recent studies suggest that some D-amino acids, such as D-glutamate, N-methyl-D-aspartate, D-alanine, and D-glutamine, could also function as messenger molecules [115]. In fact, D-glutamate is a component of the cell wall in some bacteria and is synthesized by a pyridoxal 5′-phosphate (PLP)-dependent glutamate racemase [116,117]. Furthermore, the levels of D-glutamate seem to be related to the cognitive functions of individuals with AD or mild cognitive impairment [118]. Various pieces of evidence have linked the plasma levels of D-glutamate with the extent of cognitive decline. Consequently, patients with AD or mild cognitive impairment presented lower levels of D-glutamate in plasma, which in turn correlates with their performance on cognitive assessment tools such as the MMSE or the Alzheimer’s Disease Assessment Scale Cognitive Subscale (ADAS-cog) [118,119]. It seems clear that an appropriate level of D-glutamate is needed for adequate cognitive performance, and a decrease in its concentration could lead to cognitive impairment.

Although dietary intake of glutamate is possible (e.g., coffee, cheese, fish, vegetables, and fruits, among others), GM has an important role to play in the presence of this neurotransmitter due to the ability of different species to synthesize it. Some bacteria with the ability to synthesize glutamate during food fermentation are: *Coryneform* bacteria, *Lactobacillus plantarum*, *Lactococcus lactis*, and *Lactobacillus paracasei* [120,121], while others are able to create D-glutamate from L-glutamate, such as *Corynebacterium glutamicum*, *Brevibacterium lactofermentum*, *Brevibacterium avium*, *Mycobacterium smegmatis*, and *Bacillus subtilis* [122,123]. As glutamate reaches the CNS, various transporters come into play, facilitating its passage across the BBB, for example, in the luminal membrane, there is a facilitative glutamate transport, whereas Na^+^-dependent glutamate transport exists in the abluminal membrane [124].

GABA, a prominent inhibitory neurotransmitter in humans, is closely associated with glutamate. Certain bacteria, such as *Lactobacillus brevis* and *Bifidobacterium dentium*, produce GABA from glutamate [125]. Moreover, the synthesis of GABA in the gut is related to the increase of GABA in the CNS [59], and a reduction in the presence of GABA-producing species might result in decreased GABA levels within the CNS. This fact has been associated with cognitive impairment and AD [126]. Correspondingly, the postmortem examination of brains from individuals with AD showed diminished GABA concentrations in the frontal, parietal, and temporal lobes [126,127].

Histamine has several roles, with its primary functions encompassing cell proliferation, its involvement in allergic responses, and its neurotransmitter function within the brain [128]. Another important role of histamine is related to its involvement in the immunologic function, and it seems that histamine could act both as a pro- and an anti-inflammatory metabolite. Hence, within the brain, histamine can induce allergic inflammation by triggering the rise of pro-inflammatory cytokines, such as IL-1a, IL-1b, IL-6, and chemokines. Conversely, its interaction with H4R receptors produced anti-inflammatory responses, particularly important in the CNS [129]. Other authors found that *L. reuteri* produces histamine (at least in vitro), which inhibits the production of pro-inflammatory cytokine TNF in monocytes (TLR-2-activated). This inhibition occurs through the activation of the histamine receptor H2, ultimately regulating the transcription of the TNF genes. Additionally, other authors have found that *L. saerimneri* has the capacity to synthesize histamine, which acts as an immunoregulatory metabolite [130].

Focusing on the relationship of histamine with AD and neurodegeneration, there are again controversial data. Increased histamine levels have been associated with the illness, attributing this connection to increased neuroinflammation as a result of an elevated level of nitric oxide [131], in opposition to a diminished level of histaminergic signaling in rats with vascular dementia [132]. Further research is required to delve into the role of histamine, along with exploring its potential use as a therapy for neurodegeneration and AD.

Serotonin is an essential neurotransmitter that has a connection with AD. A decrease in the serotonin levels in temporal and frontal lobes as well as in the cerebrospinal fluid is associated with AD [133,134]. Thus, serotonin reuptake inhibitors improve AD symptoms, indicating that serotonin plays a crucial role in the etiopathogenesis of the disease [135].

This neurotransmitter can be directly synthesized by different bacteria, such as *Corynebacterium* spp., *Streptococcus* spp., and *E. coli* (at least in cultures) [136]. Serotonin is synthesized from tryptophan, and GM is essential for the production of different tryptophan metabolites [137]. After tryptophan is produced by GM, about 90% of serotonin synthesis occurs within the enterochromaffin cells (by TPH enzymes) in the gut [138]. Although the brain also has the capacity to synthesize serotonin, the microbiota seems to have the capacity to increase the serotonin synthesis in enterochromaffin cells due to the increase in the TPH1 expression. Thus, the microbiota modulates the level of serotonin in the organism [139]. In studies carried out with germ-free (GF) rats, a decrease in the circulating levels of serotonin and in some cerebral regions, such as the hippocampus, has been shown [140].

It is noteworthy that the peripheral serotonin lacks the ability to cross the BBB. However, it can impact the central serotonin production through various pathways, such as reducing the available tryptophan, inducing the transcription of TPH1 through the production of SCFAs [141], or by activating serotonin receptors along the vagus nerve [142,143]. The observed reduction of serotonin in the brain of patients with AD supports this link between GM, tryptophan, and the disease [144], as it has been shown that an increase in tryptophan intake increases serotonin levels in the hippocampus and the frontal cortex, improving memory [145] and reducing the Aβ deposition in transgenic AD mice [146].

#### 2.2.2. Parkinson’s Disease

PD is the most common type of parkinsonism. More than 80% of people with parkinsonism suffer from this pathology. Based on epidemiological data, it is a pathology that affects about 10 million people worldwide, with approximately 1% of the affected population being adults over 60 years of age, and approximately 5% over 85 years of age. The global prevalence is 200–300 persons per 100,000 inhabitants, and the incidence is approximately 2% in all stages of life. It is a disease that affects more men than women, at a 1.5:1 ratio [148,149].

This neurodegenerative and systemic disease is related to the presence of α-synuclein deposits in the soma of neurons, which causes a loss of neurons in the substantia nigra of the midbrain and the formation of Lewy bodies [150]. This leads to the nuclear motor symptomatology that appears in this disease, such as bradykinesia, resting tremor (which usually occurs unilaterally and with a frequency amplitude of 3–6 Hz), and other balance and gait disorders. However, it also produces non-motor symptoms such as mood disorders, dementia, anosmia, and some disturbances of the gastrointestinal system, such as constipation [151].

The origin of the neurodegenerative process of PD has been extensively studied in the last decades, finding several coincident patterns in patients. It is known that there is a dysfunction of the mitochondria, which plays a fundamental role in the understanding of the pathology, along with a decrease in the activity of complex I of the electron transport chain, as well as mechanisms that cause parkinsonism modulated by MPTP (1-methyl-phenyl,6-tetrahydropyridine), formed from the synthesis of meperidine [152,153].

PD has been studied on the basis of these alterations, including those whose origin occurs in the intestine and spreads to the brain. It has been possible to observe in all patients an increase of α-synuclein in the ENS and in the appendix, which is associated with alterations of the ENS and parasympathetic nerves [154]. Similarly, bacterial overgrowth in the small intestine has been observed in these patients through breath tests. A higher prevalence of *Helicobacter pylori* and a direct relationship of the aggravation of PD symptomatology was observed [155,156].

In recent years, significant scientific effort has been made to better understand the disease, implementing advanced study mechanisms such as metagenomics. Metagenomics is the study of the function and structure of isolated nucleotide sequences, through the extraction and analysis of soil samples of crops from the environment under study. Genetic material is obtained, and the samples are sequenced. In this way, it is possible to identify the bacterial families and what function they are performing, as well as their taxonomic composition, which can be explored in all domains. The first metagenomic study performed for PD was in 2015 [157], where a decrease in bacteria of the *Prevotellaceae* family was observed, as well as a positive correlation between the abundance of the *Enterobacteriaceae* family and the motor phenotype, specifically with gait difficulties and postural imbalance. A year later, a study along the same lines was carried out, finding a greater presence of *Enterobacteriaceae* and a lower presence of *Prevotellaceae* in patients with PD [158]. Evidence was also found for the proliferation of the population of *Proteobacteria*, specifically *Burkholderiales*, *Enterobacteria*, and bacteria of the *Lachnospiraceae* and *Peptostreptococcaceae* families (see Figure 2 and Supplementary Material).

*Prevotellaceae* is a family of bacteria of the order *Bacteroidales* and has an outer cell wall containing LPS. It is a strict Gram-negative anaerobic bacterium and is not normally implicated in infections. In relation to PD, recent studies have shown a decrease of almost 80% of this bacterial family in stool samples compared to control groups [159]. This distinguished the severity of constipation affecting PD patients, with a sensitivity of almost 70%. These data were also consistent with previous studies analyzing the relationship of this bacterial family with CNS disorders, such as autism. *Prevotellaceae* is a commensal microorganism that inhabits the colon, and its functions are the degradation of plant polysaccharides, glycoproteins of the mucous layer of the intestine, and interaction with the body’s immune system [160,161].

The enterotype of *Prevotellaceae* in the microbiota indicates that there is a relationship between elevated levels of neuroactive SCFAs, the ability to biosynthesize folate and thiamine, and representation in PD patients [162]. In addition, these patients have a higher permeability of the intestine, which is consistent with a decrease in *Prevotellaceae*, which hinders mucin synthesis. Constipation is a prevalent symptom in PD, usually appearing years before motor symptoms, which complicates early diagnosis [163].

The increase of *Enterobacteriaceae* is related to the symptoms of postural instability and gait disorders (PIGD), characteristic of PD. *Enterobacteriaceae* are Gram-negative bacteria within the order Enterobacterales, and their morphology can be bacilli or cocci. They are within the group of the coliforms [163,164]. This bacterial family can reduce nitrate to nitrite, are anaerobic, and some can move by means of flagella [165].

Overpopulation of *Enterobacteriaceae* explains the motor symptomatology in PD. It is associated with postural instability, and difficulties in gait and station. The LPS that is produced by *Enterobacteriaceae* bacteria induces the creation of β amyloid. This promotes neuroinflammation of the brain by potentiating the nuclear factor kappa light chains of β-cells that are activated (NF-қB) [166,167].

As explained above, evidence has also been found in the proliferation of the *Proteobacteria* population, specifically *Burkholderiales*, *Enterobacteria,* and bacteria of the *Lachnospiraceae* and *Peptostreptococcaceae* families [168]. Recent studies have shown that patients diagnosed with PD have a higher proliferation of these *Proteobacteria* compared to control groups. Studies have identified an increase in *Clostridiaceae*, *Tissierellaceae*, *Prevotellaceae*, *Desulfotomaculum*, *Atopobium*, *Planococcaceae*, *Granulicatella*, *Staphylococcaceae*, *Roseburia*, *Paraprevotellaceae*, *Bifidobacterium pseudolongum*, and *Lachnospiraceae.* The propagation of *Proteobacteria* favors inflammation and increases intestinal permeability [169,170].

However, it is interesting that these studies have not always obtained the same results. For example, recent studies showed differences in the results in the microbiota profile of PD patients. One study found decreased *Tissierellaceae*, *Paraprevotellaceae*, *Prevotellaceae*, *Lachnospiraceae*, *Bifidobacterium pseudolongum*, and *Roseburia*, and increased *Oxalobacteraceae,* in patients with PD [171]. These results were consistent with the study conducted by Pietrucci et al. [172], where more than 20 bacterial families with an impact on pathology were identified. An increase of *Tissierellaceae* and *Paraprevotellaceae* was found in healthy individuals, along with a decrease of *Oxalobacteraceae*. However, different results were found in *Lachnospiraceae*, *Prevotellaceae*, *Clostridiaceae,* and *Staphylococcaceae*, which in the first study appeared to have higher concentration levels in patients with PD, while lower levels were found in the study by Del Chierico et al. [171]. It seems evident that alterations in the microbiota occur over the course of the disease, although it is not yet possible to determine whether this is the origin or cause of the pathogenesis of PD [171,172].

In summary, it is important to note that the overall profile of the microbiota associated with PD is still unknown. Studies along these lines have attempted to delimit the consequences of the microbial modifications found in these patients. It is still unknown whether the effects are chronic or whether they can be reversed, and whether they are directly associated with the course of the disease and the aggravation of symptoms.

One of the hypotheses regarding this pathology suggests its origin in the intestine and that it reaches the brain through the interaction between both systems via the vagus nerve [8,173]. Recent studies have shown that resection of the vagus nerve and bilateral vagotomy decrease the risk of developing PD. Animal studies have shown that mice induced with PD by intragastric rotenone developed Lewy bodies in the brain stem, in the dorsal nucleus of the vagus nerve [174,175]. However, when sectioning of the vagus nerve was performed, communication was not possible, and thus the spread of the pathology to the brainstem was prevented [176].

On the other hand, it is known that the determining marker of the pathology is the presence of Lewy bodies that develop in the ENS, and this is associated with an increase in intestinal permeability [58]. Leaky gut syndrome encourages damage to this tissue to promote the inclusion of harmful substances, and is associated with the presence of dysbiosis, which would alter intestinal permeability and thus promote the occurrence of the pro-inflammatory response [177,178]. This pro-inflammatory response becomes systemic and facilitates the spread of the disease due to the alteration of bacteria, which produce endotoxins that interact with receptors such as TLR4 [177,179]. These receptors belong to a family of proteins found in the innate immune system. Studies in mice have shown that knockdown of these receptors prevents the presence of bacterial alterations, so this is one line of research that should be further explored in an attempt to block the pathological mechanisms of PD expansion [180].

Finally, IL-10 (interleukin-10) has also been studied in relation to the inflammatory processes associated with PD. This gene encodes the anti-inflammatory cytokine IL-10. Impairment of this signaling pathway has become of interest in recent years as a key pathway to prevent the development of intestinal inflammation, and therefore in relation to pharmacological treatments in this pathology [181,182]. In its anti-inflammatory function, it is able to inhibit the synthesis of inflammatory cytokines by T lymphocytes and macrophages [183]. The presence of active microglia in the substantia nigra has been studied with regards to their role in inducing neurotoxic factors through excess production of interleukin-1β (IL-1β), interleukin 6 (IL-6), and nitric oxide.

##### Metabolites Altered in PD

Although each neurodegenerative pathology is different and presents unique clinical symptomatology, they are all characterized by high intestinal permeability and neuroinflammation. In fact, much research has indicated that PD can appear in the intestine, since a high percentage of patients with this disease present with gastrointestinal disturbances even before the onset of the most characteristic motor symptoms of the disease [184].

Several studies have shown that these patients suffer from metabolic and cellular alterations that are propagated through the vagus nerve by means of the α-synuclein protein, which is transported to the brain [185]. This protein has been found in myenteric and submucosal neurons [186]. Although the disease is not initiated in the substantia nigra, it is affected by the initial pathobiological process, i.e., by the aggregation of trigger proteins of specific processes in dopaminergic neurons that are involved in decreased glutathione levels, increased iron, neuromelanin, calcium level balance, and oxidative stress. Thus, dopamine deficiency in PD is associated with cell loss in the substantia nigra, leading to nigrostriatal degeneration [187,188].

In the study of PD, when an autosomal dominant pattern is identified, mutations in the α-synuclein protein (Ala53Thr and Ala30Pro) are present. This protein is located in the presynaptic terminals and is a precursor of the non-amyloid compound of neural plaques in other neurodegenerative pathologies, such as Alzheimer’s disease [189,190]. In addition, mutations in the DJ-1 protein are related to the autosomal recessive variant of PD and its functions are gene expression, mRNA targeting, oncogenic transformation, chaperone activity, and response to oxidative stress [191,192]. The PINK-1 gene is related to the development of early-onset PD, although its specific functions are still being studied in detail [193].

- PD and Polyphenols

The main neurodegenerative diseases have common cellular and molecular characteristics in cells. Among the most important are the deposition of protein aggregates, oxidative stress, inflammation, and neuronal apoptosis [194]. In relation to polyphenols and PD, resveratrol, oxyresveratrol, quercetin, baicalein, hesperidin, kaempferol, turmeric, and epigallocatechin gallate have been studied. In the latter case, it seems to have a protective effect on neurodegenerative pathologies associated with iron-chelating functions and modulation of protective antioxidant enzymes [195].

Resveratrol [196] is associated with the inhibition of the loss of dopaminergic neurons in animal models. It can reduce neural inflammation processes by decreasing the levels of cyclooxygenase-2 (COX-2) in TNF-α in the substantia nigra and in mRNA. In turn, the oxyresveratrol derivative decreases SH-SY5Y cell damage by increasing SIRT1 levels, as well as by negatively modulating caspase-3 expression and c-Jun [197,198].

Oxidative stress is a fundamental element in the etiopathogenesis of PD. These patients present alterations in the mitochondrial respiratory chain due to complex I inhibition [199,200,201]. A large source of reactive oxygen species (ROS) is the oxidative metabolism of dopamine, which is an essential process in dopaminergic degeneration [202,203]. At least 90% of dopamine is found in presynaptic vesicles, although a small proportion is degraded by monoamine oxidase B (MAO-B) [204,205]. Prolonged neuroinflammation is another source of ROS, with increased levels of pro-inflammatory cytokines in the CSF in these patients.

Diets rich in polyphenols such as flavonoids, as well as intervention with nonsteroidal anti-inflammatory drugs (NSAIDs), have a protective role at the onset of neurodegenerative diseases and other pathologies associated with chronological age, since they can modulate NF-қB signaling pathways, and therefore, they have notable therapeutic potential [206,207,208].

- PD and LPS

Abnormalities of the intestinal microbiota in PD patients occur frequently, as mentioned above. Studies have shown that this microbiota is altered, favoring an organic inflammatory state, although it is not yet possible to know if the production of LPS and some bacteria, as well as the genes that produce them, are the beginning or the consequence of the etiopathogenesis of PD [209,210,211]. In addition, microglial cells play an essential role in the inflammatory processes associated with neurodegenerative diseases. Chronic activation of these cells promotes neuronal damage and produces an inflammatory process with harmful long-term effects [212,213]. The neurotoxic response is called reactive microgliosis. LPS, an endotoxin of the outer membrane of Gram-negative bacteria, is involved in the pathogenesis of sepsis, a cellular inflammatory process that activates lymphocytes and macrophages [212,214]. In addition, these patients have an increased permeability of the intestine, measured by using urinary tests, among other tools [215]. This could indicate that certain toxic substances circulate freely through the microbiota–gut–brain axis, as explained above, favoring the toxicity to neurons in patients with PD.

- PD and SCFAs

Short-chain fatty acids and tryptophan metabolites perform upstream signaling trigger functions across the intestinal barrier into the bloodstream and across the BBB [8,216]. These metabolites regulate the immune system locally and at the CNS level, generating a pro-inflammatory environment. In animal models, it has been possible to observe an increase in adrenocorticotropin and corticosterone production and a decrease in neurotrophic levels, which normalize when the intestinal microbiota is balanced [144,217]. Specifically, in the study of PD metagenomics, a more inflammatory profile was observed in the mucosa of these patients [218,219]. Similarly, lower levels of hydrogen-producing bacteria and lower levels of bacterial lipopolysaccharide-binding proteins have been observed, which favor greater intestinal permeability [220,221,222].

Other studies found modifications in the number of some axons associated with a poorer cognitive profile in these patients, a worse emotional profile, and sleep disorders, as well as a relationship with the worsening of motor symptomatology [223,224]. This is relevant because the action of bacterial metabolic pathways involved in the metabolization of xenobiotics are altered, and this could have a negative impact on the specific treatment of this pathology [225].

#### 2.2.3. Multiple Sclerosis and Amyotrophic Lateral Sclerosis

As previously mentioned, other neurocognitive disorders in which GM could be involved include MS and ALS. MS is a chronical autoimmune disorder in which the human immune system attacks and destroys the myelin sheath, affecting the normal propagation of nerve impulses. Moreover, the alteration of the myelin sheath evokes neuroinflammation and abrasion formation. These alterations produce physical and cognitive impairments that eventually lead to paralysis and disruption of the BBB [226,227].

ALS is a neurodegenerative disease that is caused by a degeneration of the lower and upper motor neurons [228]. The neuronal death produces neuromuscular weakness, wasting, cramps, and fasciculations. This disease has an incidence of 1 to 2 new patients diagnosed per 100,000 a year in the EU and Europe [229,230].

In terms of dysbiosis, significantly fewer operational taxonomic units (OTUs) classified as *Bacteroidaceae* and *Faecalibacterium*, and a higher number of *Ruminococcus* OTUs have been found in MS patients compared to healthy controls. Especially relevant is the fact that *Faecalibacterium*, associated with a reduced inflammatory state due to its role in butyrate production, appears to be less abundant in MS patients overall [231]. SCFA production has been shown to be neuroprotective, and some of the beneficial bacteria responsible for its production, such as Prevotella, are decreased in pathological conditions such as multiple sclerosis [232].

Other studies have found significant increases in the relative abundance of *Bacteroidetes*, and a significant reduction in *Firmicutes*, in ALS patients compared to controls. Furthermore, the authors observed a decrease in gene function associated with metabolic pathways in ALS patients, and demonstrated discrepancies in microbiota at the species level and in relevant metabolites (see Figure 2 and Supplementary Material) [233,234].

Intestinal barrier dysfunction plays a key role in inflammatory gastrointestinal pathologies and neuropathologies. Cells of the central neuronal system, astrocytes and microglia cells, are regulated by different metabolites originating from intestinal symbiotics. The pathway of these chemicals inhibited neurodegeneration and neuroinflammation in models of experimental autoimmune encephalomyelitis (EAE). There is solid evidence to support the idea that the gut microbiome as well as small-molecule metabolites from the gastrointestinal tract play an essential role in the pathogenesis of ALS when they reach the central nervous system via the BBB. In an animal model of ALS, with G93A-SOD1 transgenic mice, a clear involvement of tight junctions and the intestinal epithelium in the progression of ALS was found. Likewise, there was evidence that probiotic ingestion and replenishment of essential metabolites improved motor skills in these mice [235]. After a slow reduction in the levels of *A. muciniphila* with the progression of the disease in these mice, treatment with *A. muciniphila* favorably modulated the course and severity of the disease [236].

Likewise, an imbalance between potentially protective microbial groups, such as *Bacteroidetes*, and others with potential neurotoxic or pro-inflammatory activity, such as *Cyanobacteria*, has been shown [237].

As previously mentioned, the microbiota also regulates immune cells in the gut and is important in the metabolism of essential amino acids, such as tryptophan, phenylalanine, and branched-chain amino acids (leucine, isoleucine, and valine). Despite the high interindividual variability in the composition of the microbiota, its metabolic effects are more stable, suggesting that these metabolic functions may be fulfilled by different microbial communities in the hosts [232,237,238].

Metabolomic stability versus microbiome variability invites us to focus on the characterization of the metabolome in health and disease states, based on the hypothesis that some gut metabolites can be transferred to the CSF, where they might have a toxic effect and thus impact the disease course. Based on these findings, it is plausible to consider that the altered balance between bacteria with beneficial and detrimental effects may lead to depletion of neuroprotective metabolites, parallel to a potential accumulation of neurotoxic compounds [232,239].

Both MS and ALS are associated with metabolomic changes. Metabolomics may not only have the ability to distinguish MS and ALS patients from other neurological diseases, but also may allow a better insight into the pathophysiology of these diseases, as well as the building of predictive models to stage the diseases and monitor progression through diagnostic and prognostic biomarkers [234,240].

There is a clear need to identify biomarkers for both the diagnosis and the progression and prognosis of MS, which is critical during the later stages of the disease [241]. In recent years, metabolomics has positioned itself as a useful tool in the identification of possible biomarkers in MS, which provides important information for the integration of individualized interventions that may support an improved response to treatment [242].

Targeted metabolomic analysis of plasma and CSF samples from healthy controls and patients with MS and ALS may be useful to identify neurotoxic metabolites. Studying metabolic pathways and identifying consistent metabolic abnormalities may help establish potential targets for therapeutic intervention [239,243].

##### Metabolites Altered in MS and ALS

- Multiple Sclerosis

The microbiota has been linked to myelin synthesis regulation in MS in GF mice. Gut–brain communication may be mediated by microbially derived metabolites, such as phenol and indole derivatives of tryptophan and phenylalanine catabolism, and may induce neurotoxicity in MS [244,245]. These metabolites can reach the CNS through the bloodstream and the CSF, and the abundance of neurotoxic metabolites in CSF correlates with biomarkers of neurodegeneration and brain volume. It is important to highlight that increased levels of LPS and LPS-binding protein in the plasma of MS patients are associated with increased levels of pro-inflammatory cytokines, leading to deterioration of the integrity of the BBB and perpetuating the pathogenicity of MS [246].

- MS and Tryptophan

Certain tryptophan derivatives in multiple sclerosis have been shown to directly bind to the aryl hydrocarbon receptor on glial cells, where they have an anti-inflammatory effect [232]. Metabolomic evaluation has shown lower levels of tryptophan in MS patients compared to controls [242,244]. Differences in tryptophan metabolism have also been found in progressive multiple sclerosis compared to controls or relapsing–remitting MS (RRMS) [243,247].

Although tryptophan can also be metabolized towards serotonin, the predominant pathway, responsible for up to 95% of its metabolism, is the kynurenine pathway (KP), and the first major findings regarding the KP in MS patients, corroborated in several other studies, was that tryptophan levels are significantly lower in the CSF and the serum of these patients [248].

A central role of kynurenine in the pathogenesis of MS has been well-established, related to the neurodegenerative process, both in animal models and in human subjects. The lower levels in the CSF and serum of MS patients implies measurable activation of the KP during the disease. Strategies aimed at rebalancing the KP could be helpful therapeutic approaches in slowing neurodegeneration in MS [248,249].

Some evaluations have proven that kynurenic acid (KYNA) levels are elevated during acute relapses, while they are lower in the remission phase. Lower rates of KYNA have also been found in progressive disease courses and RRMS patients compared to healthy controls [232]. KYNA is a potent antioxidant of the central nervous system, and both in vivo and in vitro evidence shows that KYNA can firstly halt lipid peroxidation, and secondly act as a scavenger for ROS, which further increases its neuroprotective capabilities [248,250,251]. Based on the neuroprotective role of KYNA in MS, it has been included as a predictor in a highly sensitive (up to 91%) model for the prediction of the disease course [252].

Quinolinic acid (QUIN) has also been evaluated as a useful neuroinflammation and neurodegeneration biomarker in the CSF of RRMS patients. Along with neurofilaments and neopterin, QUIN is elevated in the serum of MS patients compared to controls and is one of the best predictors of disease severity [253].

The upregulation of the enzyme kynurenine 3-monooxygenase (KMO) in EAE rats leads to QUIN production at higher and neurotoxic levels in the spinal cord. On the contrary, its inhibition results in decreased levels of neurotoxic molecules and elevates KYNA levels [254,255]. High KMO activity has been found in perivascular, subependymal, and subpial macrophages in EAE rats, meaning that the peripheral cells allowed by the BBB contribute to the source of neurotoxic kynurenine metabolites [256].

Chronic exposure of the rat striatum to QUIN or in vitro treatment of human neurons with pathophysiological doses of QUIN induced cognitive dysfunction and apoptosis, respectively [257,258].

QUIN harshly disrupts mitochondrial functioning, inducing neurodegeneration and cell death, since it may inhibit the neutralization of ROS and other free radicals in the cells, change the glutathione redox potential, and deplete superoxide dismutase activity [259,260]. Therefore, QUIN is a potent neurotoxin, produced in macrophages, and to a lesser extent in microglia, and both cells are hallmarks of MS lesions in the active phase due to BBB damage, which allows for the invasion of macrophages into the CNS [261].

It is relevant that both macrophages and microglia steadily release pro-inflammatory cytokines, what leads to increased QUIN and other toxic KP metabolites. In this way, neuronal injury and death occur as a consequence of the positive feedback loop that becomes established [262].

Indoxyl sulfate (IS) is derived from the breakdown of tryptophan by colon microbes, and its relative abundance has been found to be higher in the CSF of RRMS and secondary progressive MS (SPMS) patients compared to controls. This metabolite seems to be sufficient to decrease neuronal function, due to its neurotoxic effect, in cultured neurons, and this is not dependent on impacting mitochondrial respiration or oxidative stress [263]. IS has been validated as a neurotoxic compound due to its significant correlation with the levels of NFL, a well-accepted biomarker of neurodegeneration, in the CSF [232,264].

- MS and Phenols

For phenylalanine catabolism/derivatives, various studies have shown a significant decrease in phenylalanine in MS patients compared to controls, in addition to differences in its metabolism between patients with progressive MS and RRMS [243,263,264]. Phenol-derived metabolites directly impair neuronal physiological function, and chronic exposure of cultured neurons to increasing concentrations of these metabolites has been shown to lead to a dose-dependent neurotoxic effect, consisting of induced axonal damage; that is, independent from mitochondrial dysfunction and oxidative stress, thereby representing a novel pathway of neurotoxicity.

The microbiota–gut–brain axis has been shown to regulate myelin-related genes and myelination processes. Specifically, microbial-derived phenolic compounds, such as 4-ethylphenyl sulphate and p-cresol, induce dysfunction in the myelination of neuronal axons [265,266], which could be of great relevance not only for MS but also for other neurodegenerative diseases. Other results that support these findings have revealed that the levels of these phenol metabolites (e.g., p-cresol sulphate and N-phenylacetylglutamine) inversely correlate to MRI measurements of cortical volume and directly correlate to neurofilament light-chain levels [232].

- MS and Branched-Chain Amino Acids

Metabolomic evaluation has shown higher levels of lactate and lower levels of valine and tyrosine in MS patients compared to controls. A metabolomic imbalance with mitochondrial dysfunction has been detected by higher lactate levels and lower levels of tryptophan, tyrosine, and valine in MS patients, compared to healthy controls [244]. As biomarkers, glucose and valine allow distinguishing MS and controls, and other metabolites allow distinguishing between MS and another inflammatory demyelinating disease, neuromyelitis optica (NMO). Specifically, scyllo-inositol and glutamine are higher in MS, and acetate, glutamate, lactate, and lysine are higher in NMO [243]. Another analysis to characterize the relapse and remission status showed that isoleucine and valine were downregulated in MS relapse compared to MS remission [267].

- Amyotrophic Lateral Sclerosis

Growing evidence indicates that the gut microbiome may actively contribute to ALS pathogenesis. A shift of the microbiome profile towards reduced beneficial bacteria and elevated intestinal inflammation has been reported in ALS, both in experimental animal models and human studies, and even repeated antibiotic usage, which has a considerable impact on the microbiota, has been associated with an increase in the risk of ALS [268]. There are different ALS animal models that involve dysbiosis. Moreover, the symptoms could be modified or modulated by using different metabolites or antibiotics. These results have shown that the GM has the capacity to modify the motor deficits or neuroinflammation observed in ALS [269,270].

The neuroinflammation in ALS contributes to making the disease worse via different mechanisms, such as the increase of ROS, the increase of immune system activation, or the increase in the release of pro-inflammatory cytokines [236]. Moreover, this inflammation occurs first in the gut, then at a systemic level [240].

Transgenic ALS animals have an altered GM. For example, G93A mice have lower amounts of species such as *Butyrivibrio fibriosolvens*, *Escherichia coli*, and *Fermicus*, as well as bacteria that produce butyrate, compared to control mice. This indicates that the GM may have an important role in ALS onset and evolution [236].

Patients with ALS also have different GMs than healthy controls. The 16s rDNA technique revealed that patients with ALS have more *Bacteroidetes*, while *Firmicutes* and *Megamonas* are diminished, compared to healthy controls [271,272]. It has been shown that γ-glutamyl phenylalanine could be considered as a risk factor for developing the disease, and patients with ALS have also shown a lower level of gene function in several metabolomic pathways [273].

Another study analyzed stool samples from ALS patients, concluding that, as in previous studies, ALS patients showed a loss of diversity in their GM. Moreover, they found that majority of patients had high intestinal inflammation (measured by the presence of metabolites such as LPS) [274]. Therefore, the use of prebiotic, probiotic, or metabolite treatments (for example, *Prevotella* spp., or changing butyrate metabolism) could be useful in the treatment of ALS [275].

- ALS and SCFAs: Butyrate

As previously mentioned, the production of SCFAs by the microbiota affects various processes, such as the absorption of nutrients, and plays a key role in energy metabolism. Butyrate (one of the main SCFAs) seems to have a role in the reduction of the accumulation of mutated proteins, not only in an ALS mouse model, but also in human intestinal epithelial cells. Moreover, dysbiosis reduces the diversity of bacteria that produce butyrate in animal models of ALS [234], and a recent study found differences in 15 species in patients with ALS and healthy controls, including some of those that produce butyrate [276].

In an ALS mouse model, the reduction in the GM of butyrate-producing bacteria (for example, *Butyrivibrio fibrisolvens*, *Roseburia intestinalis*, or *Eubacterium rectale*) was observed two months before the onset of the disease. The total amount of butyrate was reduced in ALS patients compared to healthy controls [236,274]. Finally, the use of sodium butyrate as a treatment helped to reduce the ALS symptoms in the mice. Thus, mice treated with sodium butyrate had better health than those who did not receive the treatment. Specifically, treated animals showed better intestinal barrier function and a delay of 50 days for weight loss and 38 days for death [254].

- ALS and Tryptophan

Other metabolites that are altered in ALS are those related to the tryptophan–nicotinamide (NAM) pathway. For example, the level of NAM in CSF seems to be lower in ALS patients than in healthy controls. The microbiota has the capacity to alter this pathway by producing different metabolites. Some species from GM produce tryptophan, which plays a key role in the activation of astrocytes and microglia. These results suggest that some metabolites synthesized by the microbiota could have properties relevant to the regulation of the neuroinflammation observed in ALS patients or may even help to control some symptoms that are present in ALS, such as non-motor cognitive and behavioral alterations [236,277,278].

Other GM metabolites that are downregulated or upregulated in ALS patients include NO and GABA, and as in other neurodegenerative diseases such as AD, LPS, which is increased in the colon and small intestine of ALS mice [236]. LPS may alter gut homeostasis, inflammation, and permeability, activating several immune cells to produce pro-inflammatory cytokines, which cross the BBB and lead to neuroinflammation and neuronal death [279,280].

## 3. Interventions That Improve Symptomatology through the Intestinal Microbiota

**Figure 3:**
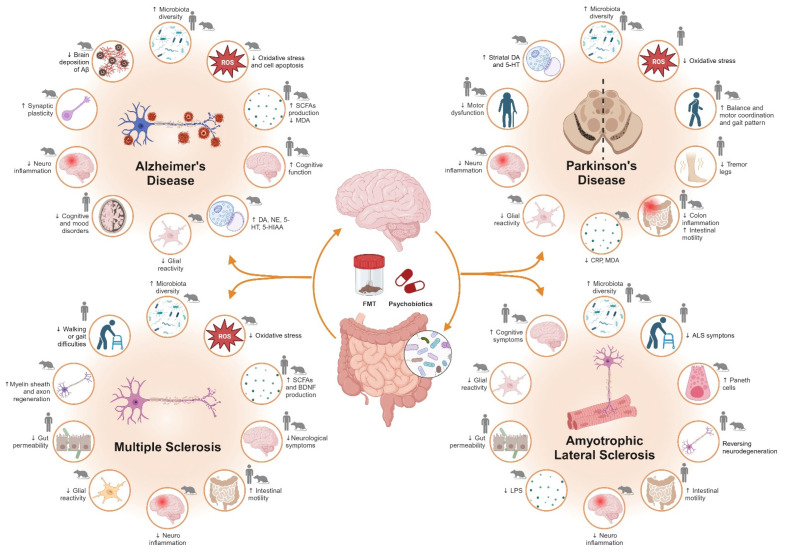
Benefits of gut microbiota interventions in neurodegenerative diseases. Beneficial effects of fecal microbiota transplantation and the use of psychobiotics (pre- and pro-biotics) in AD, PD, MS, and ALS in animal and human studies. Arrows represent increase or decrease compared to healthy controls, according to the literature. Abbreviations: 5-HIAA: 5-hydroxyindolacetic acid; 5-HT: 5-hydroxytryptamine (serotonin); Aβ: amyloid beta protein; ALS: amyotrophic lateral sclerosis; CRP: C-reactive protein; DA: dopamine; FMT: fecal microbiota transplantation; LPS: lipopolysaccharides; MDA: malondialdehyde; NE: norepinephrine; ROS: reactive oxygen species; SCFAs: short-chain fatty acids.

Since significant differences in the composition and abundance of the gut microbiota have been found between healthy individuals and those with neurological disorders, and it was possible to confirm the influence of the gut microbiome on metabolism. Microbial dysbiosis plays a key role in the pathogenesis of common neurological disorders, and strategies aimed at modulating the gut microbiota are showing great therapeutic potential [281]. Multiple factors influence the composition of the microbiota, such as diet and physical activity. However, this review focuses on those strategies that directly provide the subject with controlled microbial populations, thus seeking to reprogram the subject’s microbiome (see Figure 3).

The microbiota can be modulated through different mechanisms of action. Prebiotics are “a substrate (carbohydrate-based polyphenols and polyunsaturated fatty acids) that is selectively utilized by host microorganisms, conferring a health benefit” [282]. Thus, prebiotics help to maintain a healthy microbiota by enhancing the action of bacteria that are in the gastrointestinal system. Probiotics are live microorganisms that can be orally administrated as a food supplement or medicine (such as *Lactobacillus* and *Bifidobacterium*, which are the most used [283]), and that, when used in the proper amount, confer health benefits to the host [284,285]. Postbiotic products have been defined as those substances produced by probiotic microorganisms, which have beneficial effects for the organism. Psychobiotics include prebiotics and probiotics that, once ingested, improve the CNS symptoms associated with psychiatric and psychological disorders [286].

Fecal microbiota transplantation (FMT) is a method for reprogramming the gut microbiome, transferring fecal material from a healthy donor to a recipient, and aiming to normalize the composition and function of intestinal microbiota populations. FMT is the transfer of gut microbiota from a “healthy” individual into the gastrointestinal tract of a diseased individual with the aim of correcting dysbiosis in the recipient [287], and it is currently available in three forms: nasal and intestinal transplantation, oral capsule transplantation, and an endoscopic spray.

FMT has been used to treat infection with *Clostridium difficile* for nearly 60 years because of its remarkable effectiveness and it is increasingly used to treat other gastrointestinal diseases [288]. FMT has shown a nearly 90% success rate for the treatment of recurrent *Clostridium difficile* infection, with expansion of its application to the treatment of other gastrointestinal disorders [289,290]. Recently, interest in extending FMT to other pathologies associated with gut dysbiosis has grown, particularly for functional and neurodegenerative diseases [291].

According to the WHO (World Health Organization) and FAO (Food and Agriculture Organization of the United Nations), probiotics are products derived from live microorganisms that, if ingested in adequate amounts (>10^6^–10^8^ colony-forming units (CFU)/g or >10^8^–10^10^ CFU/dose of viable cells), provide beneficial effects to the host. Probiotics are often used with the aim of restoring homeostasis; for example, after antibiotic treatment, which can cause alterations in the microbiota [292].

During the last 15 years, the interest of the scientific community in the study of probiotics and their role in modulating the functioning of the CNS has significantly increased, in addition to interest in the moderation of cortisol levels through the interaction of neurotransmitters in the vagus nerve and the nerves of the ENS, in brain-derived neurotrophic factor (BDNF) or neurotrophic factor systems, in behavior and cognitive function through the chemistry of the CNS, in the immune system, and in the endocrine system [293,294].

### 3.1. Alzheimer´s Disease

#### 3.1.1. Fecal Microbiota Transplantation

Several studies have used FMT to demonstrate the involvement of the gut microbiome in AD pathogenesis, progression, and severity, using well-characterized mouse models of the disease. For example, FMT from 5xFAD mice into wild-type (WT) recipients significantly impaired memory function, decreased hippocampal neurogenesis, and increased inflammation in the brain and colon of the recipients [295]. Fujii et al. created a humanized mice model, observing that FMT from a human AD patient into WT mice remarkably affected mouse behavior. Additionally, lower metabolites related to the nervous system (i.e., γ-aminobutyrate, taurine, and valine) were noted in the feces of these animals [296]. Dodiya and colleagues, using an APPSWE/PS1L166P (APPPS1-21) mouse model of Aβ amyloidosis, demonstrated that FMT from age-matched APPPS1-21 male mice without antibiotics into antibiotic-treated APPPS1-21 male mice reinstituted the gut microbiome and partially restored Aβ pathology and microglial morphology [297]. Similarly, Wang et al. (2021) treated 3-month-old APPSWE/PS1ΔE9 mice with antibiotic cocktails prior to FMT from 16-month-old APPSWE/PS1ΔE9 mice and showed a significant increase in Aβ plaques in the recipient mice [298].

Recent studies, conducted primarily in mice but also in humans, have found that FMT positively affects AD subjects. Sun et al. (2019) performed FMT from WT mice into APPSWE/PS1ΔE9 transgenic mice, producing a significant improvement of cognitive deficits and reducing the brain deposition of Aβ. Additionally, they observed that FMT treatment increased synaptic plasticity, mitigated neuroinflammation, and reversed the changes of the gut microbiota and SCFAs (butyrate) [299]. FMT from WT mice into another transgenic mouse model of AD (ADLPAPT mice) ameliorated the formation of Aβ plaques and neurofibrillary tangles, glial reactivity, and cognitive impairment [300]. Furthermore, in an interesting study, old (30- to 32-week-old) 5xFAD mice were treated with FMT from either young (10- to 12-week-old) or age-matched (30- to 32-week-old) WT donor mice. Improvements in spatial memory and learning, and a significant reduction in amyloid plaque load, in the old 5xFAD recipient mice was observed. Moreover, old 5xFAD mice that received FMT from young donors showed the most robust changes, highlighting the crucial role of donor age in FMT efficacy [301].

In human studies, two case studies showed promising results. In the first study, an 82-year-old male patient received FMT from his 85-year-old wife, to treat recurrent *Clostridium difficile* infection. Improvements in AD symptoms (mood, memory, and cognitive function) occurred as early as 2 months after FMT and persisted at the 6-month follow-up visit [302]. The second case study involved a 90-year-old woman with AD and severe *Clostridium difficile* infection who underwent FMT from a healthy 27-year-old man (donor). An improvement in cognitive function, microbiota diversity, and SCFA production was observed [303].

#### 3.1.2. Prebiotics and Probiotics

Studies in animal models have shown the benefits of the use of probiotics for the improvement of behaviors associated with certain psychopathologies, such as obsessive-compulsive disorder (OCD) and autism spectrum disorder (ASD), as well as in higher abilities such as memory and attention. However, there is still little evidence regarding the use of prebiotics and probiotics in AD [304,305,306].

Recent studies have shown that these products modify the intestinal microbiota and improve cognitive functioning through the expression of neurotransmitter receptors and neuromodulators [307]. Specifically, studies in animal models showed inhibition of oxidative stress and cell apoptosis. It has also been verified that it improves metabolic processes by restoring the mitochondria membrane potential [8,308].

Studies using the oligosaccharide *Morinda officinalis* (OMO) in an animal model showed that the group treated with this product had better cell morphology, as well as higher levels of dopamine, noradrenaline, 5-hydroxytryptamine (5-HT), and 5-hydroxyindole acetic acid (5-HIAA). The group that ingested the product showed inhibition of malondialdehyde (MDA) production, which has been associated with the mechanisms of neurodegeneration in AD. In addition, OMO increases the levels of anti-inflammatory cytokines [309,310,311].

Other studies in animal models have addressed the mechanisms of action of inulin, which produces short-chain fatty acids and is able to reduce oxidative stress [312,313]. A study by Pistollato et al. (2016) showed that inulin intake facilitated the proliferation of Prevotella, which is associated with the production of short-chain fatty acids. This same study showed that this product is associated with an increase of the metabolite scyllo-inositol in the hippocampus and a decrease of myo-inositol, which has been associated with demyelination and glia cell proliferation [314].

Regarding probiotics, studies in relation to AD have observed the impact of decreased dysbiosis. Ingestion of a dose of 15 × 10^9^ CFU of *Lactobacillus acidophilus*, *Bifidobacterium bifidum*, and *Bifidobacterium longum* improved memory and plasticity in rats. Intervention with *Lactobacillus acidophilus*, *Lactobacillus fermentum*, *Bifidobacterium lactis*, and *Bifidobacterium longum* in AD rats improved memory and learning, as well as reduced the size and number of Aβ plaques [315,316].

On the other hand, *L. plantarum* is related to the production of metabolites involved in chemical mechanisms in the brain, in addition to functioning as a neuronal protective factor. Specifically, strain MTCC1325 produces acetylcholine in the hippocampus and cerebral cortex, which explains the improvement in behavior and memory [317].

In humans, the first study on AD and the use of probiotics used *Lactobacillus acidophilus*, *Lactobacillus casei*, *Bifidobacterium bifidum*, and *Lactobacillus fermentum* at doses of 2 × 109 CFU/g and evaluated the effects on cognitive function using the Mini Mental State Examination. The researchers found an improvement in the evaluated functions, as well as a reduction of MDA [315,317,318,319] (see Figure 3).

### 3.2. Parkinson’s Disease

#### 3.2.1. Fecal Microbiota Transplantation

Among the findings showing that gut microbial changes play an important role in the induction and progression of PD are studies on FMT. Sampson et al. (2016) revealed that FMT from PD patients into genetically susceptible ASO (alpha-synuclein-overexpressing) mice, a mouse model of PD, enhanced motor impairment compared to mice that received FMT from healthy donors. Additionally, an altered SCFAs profile was found in mice receiving FMT from PD patients, with a lower concentration of acetate, but increased propionate and butyrate [320].

There are several animal studies supporting FMT as a promising alternative therapy for PD. In MPTP (1-methyl-4-phenyl-1,2,3,6-tetrahydropyridine)-induced PD mice, Sun et al. found that FMT from healthy mice alleviated physical impairment and reduced gut microbial dysbiosis, increasing the abundances of *Firmicutes* and *Clostridiales*, while reducing the abundances of *Proteobacteria*, *Turicibacterales,* and *Enterobacteria*. FMT decreased fecal SCFAs and increased striatal DA and 5-HT levels in PD mice [321], as well as inhibited the activation of microglia and neuroinflammation [321,322]. Increased DA levels were also found in MPTP-induced PD mice, pretreated with an antibiotic cocktail, that had received FMT from normal mice with fasting, mimicking the diet treatment [323]. Recently, another study in MPTP mice reported that FMT from normal healthy mice significantly improved the motor dysfunction in PD mice. Moreover, FMT decreased inflammation in the colon as well as in the substantia nigra and reconstructed the composition of the gut microbiota in MPTP mice [324].

In humans, the first case study on FMT to treat PD was conducted in a 71-year-old male patient with PD and constipation, with stool obtained from a 26-year-old male. The symptoms of constipation were improved after FMT. The tremor in his legs almost disappeared 1 week after FMT treatment, but gradually reappeared in the right lower limb after 2 months [325]. In a case series published by Segal et al. (2021), six patients with PD and constipation were treated with FMT, with motor, non-motor, and constipation improvements reported in five of the patients four weeks after FMT. Only one of the patients had adverse side effects [326]. Similar results regarding motor, non-motor, and constipation symptoms were found in a study with 11 PD patients [327], along with observation of an increased abundance of *Blautia* and *Prevotella* after FMT, while the abundance of *Bacteroidetes* significantly decreased.

In a study carried out by Xue et al. (2020), 15 PD patients showed improved motor and non-motor symptoms 1 month after FMT. Ten of the patients received FMT via colonoscopy and five received FMT via nasal-jejunal tube. Three patients refused to continue at the three-month follow-up, and the adverse events were mild (five cases). Interestingly, colonic FMT showed significant improvement and longer maintenance of efficacy compared to naso-intestinal FMT, highlighting the importance of the route of administration in FMT efficacy [328]. Recently, a double-blind, placebo-controlled pilot study, using orally administered, lyophilized FMT twice weekly for 12 weeks, reported improvements in subjective motor and non-motor symptoms, gut transit, intestinal motility, and constipation in PD patients. Moreover, proportions of selective families within the phylum *Firmicutes* significantly increased after FMT, while the proportion of *Proteobacteria* was significantly reduced [329].

#### 3.2.2. Prebiotics and Probiotics

Recent studies have demonstrated the positive effects of the intake of these products on the symptomatology associated with PD, although the literature is currently limited. Specifically, Tamtaji et al. (2019) studied the response to the administration of a probiotic containing *Lactobacillus acidophilus*, *Bifidobacterium bifidum*, *Lactobacillus reuteri*, and *Lactobacillus fermentum*. After intake for 12 weeks, significant differences were observed between the control group and the group of participants who took the probiotic. The concentrations of C-reactive protein and malondialdehyde decreased, and glutathione levels increased, thus improving the level of oxidative stress. The patients who received the probiotic had better scores on the Movement Disorders Society–Unified Parkinson’s Disease Rating Scale, a unified scale for the assessment of symptoms associated with PD, after the intervention [330].

Another symptom that improves after ingestion of a probiotic in combination with prebiotics is constipation, a common gastrointestinal symptom in PD. A study in an animal model that evaluated the neuroprotective function of probiotics in the dopaminergic neurons of the substantia nigra showed improvements in motor functioning in those mice that took the probiotic. Specifically, the neuroprotective effects led to improvements in balance, motor coordination, and gait pattern [330].

Regarding cognitive dysfunction, a study by Jia et al. in 2016 observed the neuroprotective role of chitosan oligosaccharide (COS), which is obtained from the shells of certain crustaceans. The oxidative stress inhibition capacity of chitosan showed positive results, decreasing the blood lipid levels. In addition, higher levels of superoxide dismutase and glutathione peroxidase enzyme activity were found, leading to a reduction of oxidative damage. COS also inhibited the inflammatory response by reducing the release of pro-inflammatory cytokines [331].

These results provide a starting point for alternative interventions. However, it is necessary to further study the effects of probiotics and prebiotics in PD. The studies carried out to date have usually analyzed the effect of these products on the digestive symptomatology associated with the pathology, affirming their neuroprotective and neuro-modulatory effects in the different systems of the organism [332] (see Figure 3).

### 3.3. MS and ALS

#### 3.3.1. Fecal Microbiota Transplantation

Different animal studies have explored the use of FMT in MS, principally using the experimental autoimmune encephalomyelitis (EAE) model, which can simulate the clinical manifestations and pathophysiological characteristics of MS. Berer et al. (2017) transferred microbiota from human MS-discordant monozygotic twin- or healthy twin-derived microbiota to a transgenic mouse model of spontaneous brain autoimmunity, observing that gut microbiota from MS-affected twins induced a significantly higher incidence of CNS-specific autoimmunity than microbiota from healthy twins [333]. In another study, FMT from MS patients into germ-free mice produced more severe symptoms of EAE compared to mice that received FMT from healthy controls [334]. Surprisingly, Liu and colleagues found that FMT from EAE mice (at the peak of EAE disease) into naive mice, treated to induce EAE, ameliorated disease in the recipient animals as compared to FMT from healthy naive mice. This effect was micro-RNA (miR)-dependent. Specifically, they showed that miR-30d was enriched in the feces of peak EAE and untreated MS patients. Thus, the increase in miR-30d induced an increase in the abundance of *Akkermansia muciniphila*, which increased the regulatory T cells’ ability to suppress EAE symptoms [335].

Other studies have used FMT as a therapeutic approach to MS treatment. Li et al. (2020) found that FMT from normal control mice into mice with EAE rectified, to a certain degree, the altered gut microbiota in EAE mice. Furthermore, FMT-treated mice showed alleviated clinical symptoms, delayed onset of EAE, reduced activation of microglia and astrocytes, and protection of the BBB, myelin, and axons [336]. Similar clinical results were observed by Wang et al. (2021), along with a decreased number of infiltrating cells in the spinal cords and regulated gene expression to improve inflammation in FMT-treated EAE mice [337].

Several case studies in MS patients have been published. Borody et al. (2011) reported observations of three MS patients that received FMT for constipation. In a study involving these three patients, a 30-year-old man underwent five FMT infusions, with positive results for his constipation and MS (at least 15 years without relapse). Two patients with ‘atypical’ MS, a 29-year-old male and an 80-year-old female, received 10 and 5 FMT infusions, respectively. Progressive improvement in neurological symptoms was observed after FMT and maintained for at least three (male) and two (female) years [338]. Similar results were obtained in two case studies of patients with MS and recurrent *Clostridium difficile* infection: a 61-year-old female with secondary progressive MS received a single FMT, showing immediate stabilization on the Expanded Disability Status Scale (EDSS), and her Functional System scores minimally improved over the next 10 years [339]. A 52-year-old female with RRMS received lyophilized FMT orally, improving her strength and EDSS score after one year [340].

Engen et al. (2020) published a case study of a 48-year-old male with RRMS and repeated evaluations over 12 months (with two FMT interventions). FMT was associated with an increased abundance of *Faecalibacterium prausnitzii* (butyrate-producing organism) and SCFAs, as well as increased serum BDNF levels and improved gait/walking metrics [341]. Recently, Al and colleagues reported that FMT was safe and tolerable in a clinical trial involving nine RRMS patients. Despite that the study was terminated early, the authors also concluded that FMT normalized elevated gut permeability and could beneficially alter the gut microbiota of these patients [342].

There are currently no reported animal studies on ALS and FMT. However, a clinical trial is ongoing (NCT03766321) involving 42 ALS patients (28 FMT-treated and 14 placebo). The study duration will be 12 months per patient [343]. Additionally, a recent case report was published by Lu et al. (2023), in which a 48-year-old woman with ALS and moderate to severe constipation was treated with washed microbiota transplantation (WMT), an improved methodology of FMT. WMT caused an improvement of her constipation and a plateau of her ALS symptoms. Several months later, she underwent antibiotic treatment due to a scalp trauma, resulting in ALS deterioration. Rescue with WMT successfully stopped the progression of the disease, with rapid improvement. After WMT, the diversity and composition of the patient’s gut microbiota were closer to those of the healthy donors [344].

#### 3.3.2. Prebiotics and Probiotics

Treatment with the probiotic *Lactobacillus farciminis* has been shown to reduce intestinal hyperpermeability in MS. Specifically, a study carried out in an EAE rat model showed that neuroinflammatory activity was reduced, as well as the response of the hypothalamic–pituitary–adrenal system. *Faecalibacterium prausnitzii* has an anti-inflammatory effect through the production of butyrate and the release of microbial molecules with anti-inflammatory action [345,346].

The first scientific evidence in this line of research was obtained through a study of the relationship between peripheral tolerance and EAE. Mice that were vaccinated with enterotoxigenic *Escherichia coli* achieved complete recovery in cases of mild EAE. These studies showed that animals that were exposed to prophylaxis had a lower number of inflammatory infiltrates in the gray matter, and a reduction of T cell expression. Similarly, through stimulation of FoxP3+ Treg cells, the anti-inflammatory properties were enhanced by secretion of IL-4, IL-10, IL-13, and TGF-β [347,348,349].

Other studies have focused on analyzing the role of *B. fragilis* PSA, an anaerobic Gram-negative bacillus, in MS. This compound is involved in certain immune system mechanisms by regulating Th1/Th2 levels and preventing intestinal inflammation. In a study by Ochoa-Repáraz et al. (2010), previously purified PSA was administered and showed protective and therapeutic effects against EAE. These authors proposed that *B. fragilis* PSA regulates mechanisms that allow disease amelioration [350].

Studies have also been carried out with *Bifidobacterium animalis* [351] or a combination of *Lactobacillus* strains in mice with induced EAE, showing potential therapeutic effects in ALS. For example, an increase of FoxP3+ Treg, IL-4, and IL-10 was found in mesenteric lymph nodes. However, the separated probiotic strains did not produce therapeutic effects in diseased mice [352]. The effect of another probiotic combination containing *Lactobacillus*, *Bifidobacterium*, and *Streptococcus* showed that this compound works similarly to *Bifidobacterium animalis* in its mechanisms of action, inhibiting Th1/Th17 polarization through T cells [353]. 

A small number of studies have been conducted on the impact of probiotics and prebiotics in ALS. A clinical trial analyzing the impact of *L. rhamnosus* HA-114 showed that this supplement improves cognitive functioning of the hypothalamus, reversing neurodegeneration in ALS. One of the mechanisms of action of *L. rhamnosus* HA-114 may be through improving the permeability of the intestinal barrier, and thus reducing metabolic endotoxemia [354]. In a prospective longitudinal study in ALS patients treated with a probiotic supplement (a mixture of five lactic acid bacteria), the gut microbial composition was influenced by the probiotic. However, the biodiversity of the GM did not become closer to that of the control group [238].

In an interesting study carried out on G93A transgenic mice (an ALS model), treatment with 2% butyrate restored GM homeostasis, improved gut integrity, and prolonged the life span of the animals. Additionally, abnormal Paneth cells were significantly decreased in the animals treated with butyrate [355] (see Figure 3). Polyphenols are another compound with potential neuroprotective effects in ALS [356], which should be further investigated.

In summary, a number of novel interventions are currently being implemented to improve the most characteristic symptomatology of neurodegenerative diseases. The use of prebiotics, probiotics, and psychobiotics is a treatment option that is reporting significant benefits to these patients. Evidence of this can be found in the different experimental studies that have been published in the last decade, all of which aim to promote public awareness of the best tools to use in these pathologies. More specifically, they target the scientific and health community, who must take as a starting point the latest advances and empirical evidence in order to create individualized intervention proposals with sufficient guarantees of success in improving the associated symptoms.

## 4. Conclusions

The microbiota–gut–brain axis maintains an active and bidirectional communication that can be influenced by lifestyle habits (e.g., diet and physical activity), and modulated through interventions focused on the gut microbiota, including fecal microbiota transplantation and the use of pre- and pro-biotics supplementation. Although it is not possible to confirm that the alterations produced in the microbial profile are a cause or a consequence of the symptomatology of each neurodegenerative disease, the modification of the microbiota is suggested as a therapeutic target for the treatment of these pathologies.

As many of the studies on neurodegenerative diseases have been conducted during the clinical phase of the disease, it is currently difficult to determine a causal relationship relating to the function of the microbiota. However, there is evidence pointing to a role of the microbiota in the onset and progression of neurodegenerative diseases (e.g., studies of fecal microbiota transplantation have shown that altered microbiota can induce characteristic symptomatology of neurodegenerative pathologies in healthy animals).

The present review drew the following essential conclusions on the use of the microbiota as a therapeutic target in neurodegenerative processes:

- There is growing evidence supporting that the onset and progression of neurodegenerative diseases are partially modulated by the gut microbiota. Both animal and human studies in AD, PD, MS, and ALS have shown an altered composition of the intestinal microbiota and its metabolites.
- There are some common metabolomic alterations in AD, PD, MS, and ALS, such as the presence of neurotoxins (i.e., LPS), a reduction in some neurotransmitters (e.g., GABA or serotonin) and anti-inflammatories cytokines (e.g., IL-10 and IL-22), an increase of pro-inflammatory cytokines, and upregulation and downregulation of different metabolites, such as SCFAs (specifically butyrate reduction). Moreover, these alterations can be moderated through the regulation of the microbiota.
- Among the microbiota reprogramming of interventions with positive results, fecal microbiota transplantation and psychobiotics are presented as potential therapeutic tools in AD, PD, MS, and ALS.

Deeper knowledge of the gut microbiota and the mechanisms of action of its metabolites is essential to approaching treatment of neurodegenerative diseases in an integrative manner. This would mean improvement of the disease progression and symptoms, both at the cognitive and neurophysiological levels.

## 5. Limitations of Study

Although this study is a comprehensive narrative review, some limitations have been identified that need to be considered:

- This is a developing field of study. This means that in some pathologies, it is not yet possible to find many studies that consolidate the data found.
- The studies analyzed have different research methodologies, with different samples and models (human and animal). This could be a limitation for establishing representative results. However, the growing number of publications in recent years allows trends and relationships to be established.
- As indicated in the Section 4, in some cases it is not possible to determine whether microbial modifications are a cause or a consequence of the symptomatology associated with neurodegenerative pathologies. However, this interaction indicates that the microbiota–gut–brain axis may be used as a therapeutic target in these diseases.

## Data Availability

Not applicable.

## Supplementary Materials

The following supporting information can be downloaded at: https://www.mdpi.com/article/10.3390/ijms241713294/s1.
