# Supplementary material for: Gut Microbial Metabolome and Dysbiosis in Neurodegenerative Diseases: Psychobiotics and Fecal Microbiota Transplantation as a Therapeutic Approach—A Comprehensive Narrative Review
Source: Int J Mol Sci. 2023 Aug 27;24(17):13294. doi: 10.3390/ijms241713294 (PMC10487945; doi:10.3390/ijms241713294)
Supplement: Supplementary file 1 [file ijms-24-13294-s001.zip › ijms-2562371-supplementary.pdf]

Supplementary Material

Figure S1: Description of the main phyla and species altered in neurodegenerative diseases. Arrows represent increase and/or decrease compared to healthy controls, according to the literature.

Phylum	Dysbiosis in AD	Dysbiosis in PD	Dysbiosis in MS	Dysbiosis in ALS
Firmicutes	↓ *Eubacterium Clostridium*<br>↓ *Roseburia hominis*<br>↓ *Faecalibacterium prausnitzii*<br>↓ Butyrivibrio<br>↑ *Clostridium*<br>↑ *Bacillus subtilis*<br>↑ *Staphylococcus aureus*	↑ Lachnospiraceae<br>↑ *Peptostrepto-coccaceae*<br>↑↓ *Clostridiaceae*<br>↑↓ *Tissierellaceae*<br>↑ *Desulfoto-maculum*<br>↑ *Planococcaceae*<br>↑ *Granulicatella*<br>↑↓ *Staphylococca-ceae*<br>↑↓ *Roseburia*<br>↑↓ *Lachnospi-raceae*	↓ *Faecalibacterium*<br>↑ *Ruminococcus*	↓ *Megamonas*<br>↓ *Butyrivibrio fibrisolvens*<br>↓ *Roseburia*<br>↓ *Eubacterium rectale*
Bacteroidetes	↑ *Porphyromonas gingivalis*	↓↑ *Prevotellaceae*<br>↓↑ *Paraprevotella-ceae*	↓↑ *Prevotellaceae*	↑ *Bacteroidetes*
Proteobacteria	↑ *Helicobacter pylori*<br>↑ *Escherichia coli*<br>↑ *Escherichia-Shigella*<br>↑ *Salmonella Typhimurium*<br>↑ *Pseudomonas fluorescens*	↑ *Helicobacter pylori*<br>↑ *Burkholderiales*<br>↑ *Oxalobacte-raceae*		
Actinobacteria	↓ *Actinobacteria*	↑ *Atopobium*<br>↑ *Bifidobacterium pseudolongum*		
Spirochaetae	↑ *Borrelia burgdorferi*<br>↑ *Treponema pallidum*			
Chlamydiota	↑ *Chlamydia pneumoniae*			
Enterobacteriaceae		↑ Enterobacte-riaceae		
Verrucomicrobia			↑ *Akkermansia muciniphila*	
Cyanobacteria			↑ *Cyanobacteria*	
